# Assessing the effectiveness of household-level focal mass drug administration and community-wide mass drug administration for reducing malaria parasite infection prevalence and incidence in Southern Province, Zambia: study protocol for a community randomized controlled trial

**DOI:** 10.1186/s13063-015-0862-3

**Published:** 2015-08-13

**Authors:** Thomas P. Eisele, Kafula Silumbe, Timothy Finn, Victor Chalwe, Mukalwa Kamuliwo, Busiku Hamainza, Hawela Moonga, Adam Bennett, Josh Yukich, Joseph Keating, Richard W. Steketee, John M. Miller

**Affiliations:** Center for Applied Malaria Research and Evaluation, Tulane University School of Public Health and Tropical Medicine, 1440 Canal Street, Suite 2200, New Orleans, LA USA; PATH-Malaria Control and Elimination Partnership in Africa (MACEPA), National Malaria Control Centre, Chainama Hospital College Grounds, Lusaka, Zambia; Institute for Medical Research and Training, University Teaching Hospital, Lusaka, Zambia; National Malaria Control Centre, Zambia Ministry of Health, Chainama Hospital, Lusaka, Zambia; Malaria Elimination Initiative, Global Health Group, University of California San Francisco, 550 16th Street, San Francisco, CA USA; PATH-MACEPA, 2201 Westlake Avenue, Seattle, WA USA

**Keywords:** Malaria, Mass drug administration, Dihydroartemisinin plus piperaquine

## Abstract

**Background:**

Mass drug administration (MDA) and focal MDA (fMDA) using dihydroartemisinin plus piperaquine (DHAp), represent two strategies to maximize the use of existing information to achieve greater clearance of human infection and reduce the parasite reservoir, and provide longer chemoprophylactic protection against new infections. The primary aim of this study is to quantify the relative effectiveness of MDA and fMDA with DHAp against no mass treatment (standard of care) for reducing *Plasmodium falciparum* prevalence and incidence.

**Methods/design:**

The study will be conducted along Lake Kariba in Southern Province, Zambia; an area of low to moderate malaria transmission and high coverage of vector control. A community randomized controlled trial (CRCT) of 60 health facility catchment areas (HFCAs) will be used to evaluate the impact of two rounds of MDA and fMDA interventions, relative to a control of no mass treatment, stratified by high and low transmission. Community residents in MDA HFCAs will be treated with DHAp at the end of the dry season (round one: November to December 2014) and the beginning of the rainy season (round two: February to March 2015). Community residents in fMDA HFCAs will be tested during the same two rounds for malaria parasites with a rapid diagnostic test; all positive individuals and all individuals living in their household will be treated with DHAp. Primary outcomes include malaria parasite prevalence (n = 5,640 children aged one month to under five-years-old), as measured by pre- and post-surveys, and malaria parasite infection incidence (n = 2,250 person-years among individuals aged three months and older), as measured by a monthly longitudinal cohort. The study is powered to detect approximately a 50 % relative reduction in these outcomes between each intervention group versus the control.

**Discussion:**

Strengths of this trial include: a robust study design (CRCT); cross-sectional parasite surveys as well as a longitudinal cohort; and stratification of high and low transmission areas. Primary limitations include: statistical power to detect only a 50 % reduction in primary outcomes within high and low transmission strata; potential for contamination; and potential for misclassification of exposure.

**Trial registration:**

Identifier: Clinicaltrials.gov: NCT02329301. Registration date: 30 December 2014.

**Electronic supplementary material:**

The online version of this article (doi:10.1186/s13063-015-0862-3) contains supplementary material, which is available to authorized users.

## Background

The recent Zambian National Malaria Strategic Plan 2011 to 2016 calls for ambitious efforts to work towards malaria elimination, with the establishment of at least five malaria-free zones by 2015 [1]. The Zambian government, through its National Malaria Control Centre (NMCC), has successfully scaled up the main World Health Organization (WHO)-recommended malaria control interventions (for example, long-lasting insecticide-treated mosquito nets (LLINs), indoor residual spraying (IRS), and prompt effective treatment of malaria) over the past decade and is considering alternative strategies to further reduce malaria prevalence and burden [2]. While recognizing that eliminating malaria from the country may require many years to achieve, malaria elimination is a realistic milestone for Zambia in light of recent successes in reducing the malaria burden through a collaborative effort among the NMCC and its partners [2].

During the 2012 dry season (June to November) in Southern Zambia, the effectiveness of three large-scale malaria mass test and treatment (MTAT) intervention rounds were assessed with a community randomized controlled trial (CRCT) [3]. The MTAT intervention consisted of testing all individuals in intervention areas with rapid diagnostic tests (RDTs); all positive individuals were treated with artemether-lumefantrine (AL; MTAT-AL), which is the first-line drug for treating uncomplicated malaria in Zambia. The MTAT-AL intervention aimed to reduce community-wide malaria transmission by targeting persons infected with the asexual stage, and treating both asexual (blood) stage and immature sexual stage (immature gametocytes) infections of the *Plasmodium falciparum* parasite, thereby preventing onward transmission from infected individuals.

The results of the Zambia MTAT trial [3], as well as those from a recent trial of MTAT-AL in Burkina Faso [4] and Zanzibar [5], suggest that the use of MTAT-AL will likely be insufficient to achieve malaria elimination in many settings, for several important reasons. The generally short terminal elimination half-life of AL (less than three days), combined with the short duration of the treatment regimen (taken over three days), may provide little chemoprophylactic or chemo-suppressive protection against reinfection of *P. falciparum* [6]. Shifting to a longer lasting antimalarial therapy, such as dihydroartemisinin plus piperaquine (DHAp), will extend the chemo-prophylactic period and thereby help to extend the duration of transmission suppression during mass treatment interventions. DHAp has shown better efficacy compared to AL at clearing asexual blood stage malaria infections, but while DHA has a similarly short elimination half-life to AL, piperaquine has a significantly longer terminal elimination half-life of up to 63 days [7, 8].

The results from the previous MTAT trials also suggest that alternatives to the MTAT antimalarial delivery strategy are needed. One alternative strategy to avoid some important limitations of MTAT involves treating all individuals within a specified target area with an effective antimalarial, irrespective of having a parasite infection identified by an RDT, which would constitute community-wide mass drug administration (MDA) [9]. There are several potential advantages of using MDA, especially if coupled with DHAp (MDA-DHAp), over MTAT-AL. First, RDTs used for diagnosis in the previous MTAT strategy have been shown to miss up to 50 % of low-density malaria parasite infections [10], and thereby leave a substantial reservoir of infection in the community untreated, which may then sustain continued transmission. Such individuals with low-density parasite infections missed by RDTs under the MTAT will receive treatment under MDA. Second, because MDA-DHAp may also provide effective chemoprophylaxis against new infections for the entire target population (irrespective of RDT result), it is expected to provide a larger chemoprophylactic and chemo-suppressive effect to the community than limiting drug administration to only those individuals testing positive for a malaria infection by RDT [10, 11]. As a result, MDA-DHAp is potentially more effective and cost-effective than MTAT, due both to clearance of low-density infections and by prevention of new infections in the entire population [12].

An alternative to community-wide MDA is focal MDA (fMDA), which combines aspects of both MTAT and MDA. fMDA, in this trial, consists of testing all individuals in the community for parasites using RDTs and treating those that are positive. But in households where anyone had a positive RDT, all household members are treated with an effective antimalarial, such as DHAp (MDA-DHAp), irrespective of each person’s RDT result. As such, fMDA uses real-time information from RDT results to focus MDA activities to populations at greatest risk of having, as well and perhaps acquiring, an infection. Results of the 2012 MTAT-AL data analysis in this study site of Southern Province shows considerable clustering of *P. falciparum* infections identified by RDTs at the household level, suggesting that if one person tests positive, there is a higher chance (than random) that others in the house are also infected, potentially including those with low-density infections missed by RDTs. Such spatial clustering of infections in other settings has been well documented [13]. FMDA-DHAp may therefore provide a substantial advantage to MTAT-AL, in that it would clear more infections among individuals with parasite infections clustered in households, even among those with low-density infections missed by RDTs. Additionally, DHAp given to individuals in households using fMDA should provide sustained prophylactic protection for those individuals against new infections for up to two months, perhaps among those in the community most at risk of acquiring a new infection. At the same time, fMDA would limit drug exposure to those most at risk.

MDA and fMDA using DHAp represent two potential strategies to achieve greater clearance of human infection and reduction in the parasite reservoir, and both provide longer chemoprophylactic protection against new infections. The primary aim of this study is to quantify the relative effectiveness of MDA and fMDA with DHAp against no mass treatment for reducing *P. falciparum* parasite prevalence, confirmed out-patient malaria case incidence, and monthly infection incidence in areas stratified by high and low malaria transmission. It is hypothesized that two rounds of fMDA and MDA with DHAp will be significantly more effective than no mass treatment (standard of care) at reducing these health outcomes. A secondary objective of this study is to compare the cost and cost-effectiveness of the fMDA and MDA strategies with DHAp compared to no mass treatment.

Other outcomes related to the quality of program implementation will also be measured during this study to help understand why the MDA and fMDA with DHAp interventions were, or were not, found to be effective in this setting. These outcomes include: participant adherence of taking a full course of DHAp by the fMDA and MDA interventions; clearance of asexual stage parasites at day seven following the administration of DHAp; population coverage of the fMDA and MDA interventions in the study areas; and community acceptability of participating in the fMDA and MDA interventions. Measurement for these outcomes is summarized in the Methods/Design section below, with further details provided in the Additional file 1.

## Methods/design

### Study site

**Fig. 1:**
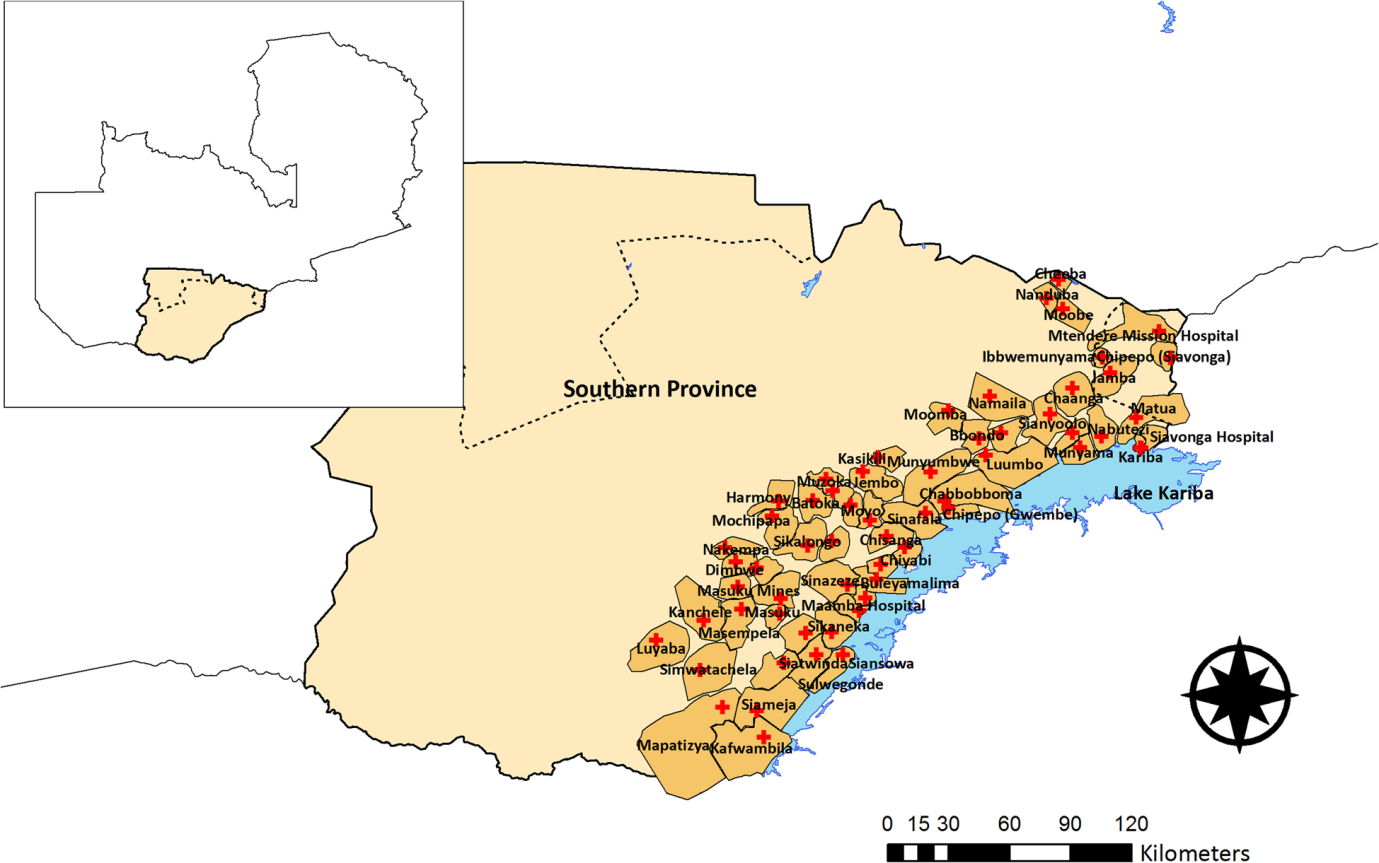
Map of the study area, divided into 60 health facility catchment areas that serve as the unit of randomization

The study will be conducted in Southern Province Zambia in districts along Lake Kariba, including Gwembe, southern Kalomo, Siavonga, and Sinazongwe (Fig. 1). The population of this area was estimated at just over 330,000 living in roughly 56,000 households in 2011, based on a complete census of the study area conducted during the previous MTAT-AL trial, plus an updated census conducted for this trial in 2014. All households in the study area were enumerated using a geographic information system (GIS) prior to February 2014.

The area is primarily composed of the Tonga ethnic group. Livelihood typically consists of fishing and farming. While there are towns in the study area, the majority of the area is rural. The health system throughout the study area consists primarily of public sector health facilities, with health facility catchment areas (HFCAs) typically covering 500 to 1,500 households. All HFCAs were mapped and geographically defined prior to this study.

Malaria transmission in this area is low to moderate, with malaria parasite prevalence ranging from less than 1 % in some areas to more than 25 % in other areas. The high malaria transmission season in the study area follows the rainy season and lasts from January to May. Results from the 2012 MTAT-AL intervention identified two distinct areas of malaria transmission intensity, with higher transmission areas along the low-lying shores of Lake Kariba, and lower transmission areas inland from Lake Kariba.

There are current efforts to maintain high vector control coverage in the study area, with household coverage of LLINs estimated to be between 60 and 80 %. Additionally, a comprehensive IRS campaign to cover all households in the study area was conducted between November 2014 and January 2015, using the highly effective insecticide Actellic.

### Interventions

#### Dihydroartemisinin plus piperaquine

DHAp is the study drug used in this trial to treat *P. falciparum* parasite infections. DHAp was recently approved as the alternate first-line treatment in Zambia for uncomplicated malaria [14]. The DHAp used in this trial was manufactured by (Sigma Tau, Gaithersburg, Maryland, USA) and is branded as Eurartesim®. Eurartesim® is the only Stringent Regulatory Authority (SRA) approved DHAp product on the international market at this time.

All individuals meeting the inclusion and exclusion criteria to receive DHAp will be offered an age-appropriate course of the drug, according to the manufacturer’s recommendations and the national guidelines. DHAp dosing will be age-based (age-based dosing table provided in Additional file 1: Table S1). Based on the treatment guidelines for uncomplicated malaria set forth by the manufacturer and the WHO, a target dose of 4 mg/kg/day dihydroartemisinin and 18 mg/kg/day piperaquine for three days, with a therapeutic dose range between 2 and 10 mg/kg/day dihydroartemisinin and 16 and 26 mg/kg/day piperaquine, will be used (please see Additional file 1: Table S1 for the age-based dosing guidelines used for administering DHAp) [4].

#### Community-wide mass drug administration with dihydroartemisinin plus piperaquine

**Fig. 2:**
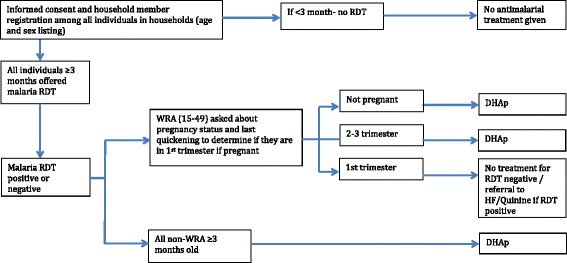
Participant flow chart to determine antimalarial treatment regimen under MDA-DHAp. Women of reproductive age (WRA): those 15 to 49-years-old; *RDT*: rapid diagnostic test; *HF*: health facility; *MDA*: mass drug administration; *DHAp*: dihydroartemisinin plus piperaquine

Under coordination with the Zambia NMCC, two rounds of MDA with DHAp are being implemented in December 2014 (end of dry season) and February 2015 (beginning of rainy season) in selected study site HFCAs. Spacing of the rounds was determined by the manufacturer recommendation of not receiving more than two courses of DHAp within two months [4]. Each round of the MDA-DHAp intervention consists of testing all individuals with an RDT, sharing the result of the RDT, and then offering an age-appropriate therapeutic dose of DHAp, irrespective of the RDT test result, among those consenting individuals meeting the inclusion criteria to receive DHAp, as outlined below and in Fig. 2. All consenting women in the MDA intervention not in the first trimester of pregnancy are offered DHAp.

#### Household-level focal mass drug administration with dihydroartemisinin plus piperaquine

**Fig. 3:**
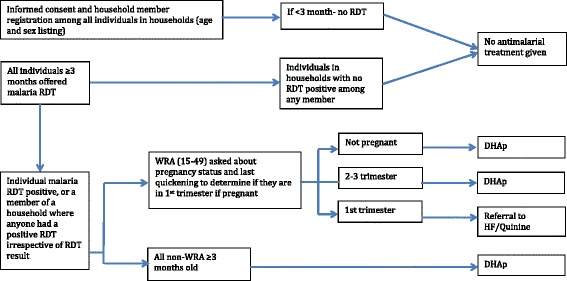
Participant flow chart to determine antimalarial treatment regimen under fMDA-DHAp. Women of reproductive age (WRA): those 15 to 49-years-old; *RDT*: rapid diagnostic test; *HF*: health facility; *MDA*: mass drug administration; *DHAp*: dihydroartemisinin plus piperaquine

Under coordination with the Zambia NMCC, two rounds of fMDA with DHAp are being implemented in December 2014 (end of dry season) and February 2015 (beginning of rainy season) in selected study site HFCAs. Each round of the fMDA-DHAp intervention consists of testing all individuals with an RDT and then sharing the result of the RDT. An age-appropriate therapeutic dose of DHAp is offered to all consenting individuals meeting the inclusion criteria to receive DHAp, as outlined below and in Fig. 3. Among all other consenting eligible individuals in the household where a positive RDT result occurred among any member, an age-appropriate therapeutic dose of DHAp is offered, irrespective of the RDT test result of that individual. All consenting women in the fMDA intervention not in the first trimester of pregnancy are offered DHAp in accordance with the fMDA process just outlined.

#### Directly observed treatment of dihydroartemisinin and piperaquine in the focal mass drug administration and mass drug administration intervention rounds

A modified directly observed treatment (DOT) will be used to maximize adherence with the prescribed DHAp treatment among all individuals participating in the MDA intervention and all individuals in households with a member with a positive RDT in the fMDA intervention. At the community level, DHAP will be given by DOT on the first day of the household visit to all household members by the campaign team testing pairs. The second day of the treatment will not be given by DOT by campaign teams, but will be asked on the final day of treatment by an adherence monitoring officer working with the campaign teams. These adherence officers will make the second household visit to administer DOT for the third treatment dose, ask about adherence for the second dose, observe and count the treatment packets for the remaining pills, and provide additional instruction on full compliance if needed.

#### Inclusion and exclusion criteria of individuals participating in the intervention

Figures 2 and 3 present an overview of the MDA-DHAp and fMDA-DHAp inclusion and exclusion criteria. Children younger than three-months-old will be excluded from receiving DHAp, according to the manufacturer recommendations. Women in their first trimester of pregnancy will not be offered DHAp in the MDA or fMDA interventions. Instead these young children and pregnant women will be offered the appropriate dose of antimalarial treatment, according to the national treatment guidelines, if found to have a positive RDT.

Pregnancy status during the first trimester will be determined among women ages 12 to 49 years through direct questioning, and by offering a urine human chorionic gonadotropin rapid pregnancy test at the field visit for women unsure of their pregnancy status or reporting longer than five weeks since last menstruation. All consenting women in the MDA and fMDA interventions with a negative RDT deemed to be in the first trimester of pregnancy will receive no malaria treatment. All consenting women deemed to be in their first trimester of pregnancy with a positive RDT will receive the recommended standard of care treatment for uncomplicated malaria in Zambia among women in their first trimester of pregnancy, which consists of age-appropriate treatment with oral quinine for seven days.

All individuals with suspected severe malaria or other severe illness (including symptoms of severe anemia, prostration, impaired consciousness, respiratory distress, convulsions, circulatory collapse, abnormal bleeding, jaundice, and passing black or brown (dark) urine) will be referred to the nearest health facility for treatment, and will not be included in the MDA or fMDA interventions or associated research.

#### Community mobilization

MDA requires high levels of participation and adherence to the full treatment course to be as effective as possible. To encourage participation, several activities were conducted in advance of and during the treatment campaigns in order to broaden and strengthen community awareness. First, information about the campaigns, the treatments, and the process were shared through the Ministry of Health and Ministry of Community Development, Mother and Child Health structures, including among community health workers to begin discussions at local levels. Second, community sensitization meetings were held throughout campaign areas across nine districts to inform leaders at the district level, including district councilors, religious leaders, and lead educators, and at the chiefdom level, with special outreach to chiefs, village headmen, local community health workers, and community members gathered for village entry meetings. Information, education, and communication materials were developed with the Zambia NMCC on malaria and pretested in campaign areas, containing behavior change communication messages for community members and community leaders. These were translated into the local language and distributed at these community sensitization meetings. Third, radio spots were developed and aired in the local language in target areas, along with a series of radio group episodes in which community members engaged with campaign health experts to learn about the program and discuss the benefits of participation.

### Standard of care and control group

Standard of care in Zambia, including the study area in Southern Province, consists of identification of malaria infections and clinical cases through passive case detection at local health facilities or community health workers. Zambian national policy stipulates that individuals seeking care for a fever be given a malaria diagnostic test by RDT or microscopy. Individuals with confirmed malaria are then treated with the first-line antimalarial in Zambia, which is AL. When no diagnostic test is available at a health facility, a clinical diagnosis is made based on signs and symptoms, and treatment follows the identical protocol as with RDT-confirmed malaria cases. Standard of care does not include any kind of active surveillance for malaria parasite infection or mass treatment of the population with antimalarials.

### Study design

A CRCT will be used to evaluate the impact of two rounds of the MDA-DHAp or fMDA-DHAp (the two intervention arms) at reducing malaria parasite infection prevalence and incidence, as well as malaria-related morbidity, compared to a control group receiving standard of care. Communities, or clusters, are defined by HFCAs and serve the unit of randomization. A total of 60 health facilities and their catchment areas are included in the study.

#### Stratification

As the impact of the MDA and fMDA interventions are expected to vary across malaria transmission strata, the 60 HFCAs included in the study have been stratified into high and low transmission strata. To do this, parasite prevalence in each HFCA was estimated with a kriged continuous surface of parasite prevalence from data from the baseline 2014 parasite survey. Catchments were then ranked from highest to lowest parasite prevalence. The top 30 HFCAs were included in the high transmission strata, while the bottom 30 were included in the low transmission strata; the resulting cut point between the high and low transmission strata was approximately 10 % parasite infection prevalence.

### Randomization sequence generation and allocation concealment

Pre-existing maps of HFCAs were used to classify households as belonging to a specific HFCA, which serve as the unit of randomization. To ensure balance across large and small HFCAs, which could potentially affect the quality of program implementation and other related unobserved confounding factors, HFCAs were characterized as having either a large or small population catchment size. This classification was based on a cut-point obtained from the mean population catchment size across the 60 HFCAs. Within the high and low transmission strata and the large and small population catchment size classifications, the random allocation rule will be used to assign HFCAs to the fMDA-DHAp, MDA-DHAp, or control group. As a result of this process and the stratification outlined above, the 60 total HFCAs are split into 10 HFCAs per treatment group (MDA, fMDA, and control) in the high transmission strata (n = 30 HFCAs), and 10 HFCAs per treatment group (MDA, fMDA, and control) in the low transmission strata (n = 30 HFCAs).

After allocation, the appropriate intervention will be implemented in the entire catchment area according to treatment group assignment. Allocation of study arms will not be blinded to the deliverers of the intervention or to the main investigators, as delivery of the intervention precludes the possibility of allocation concealment. However, data collectors in the follow-up parasite prevalence survey, as outlined below, will be blinded to allocation. All primary analyses will also be blinded to analysts by the use of concealed code for the study arm variables in the final dataset.

### Primary outcome measures

Primary outcomes used to measures the impact of the MDA-DHAp and fMDA-DHAp interventions, as compared to a control of standard of care, are:

1. Parasite prevalence during the high transmission season among children < six-years-old: defined as the proportion of children < six-years-old (≥three months to < six-years-old) with *P. falciparum* infection (based on both RDT and PCR) out of all children < six-years-old tested within the 2014 and 2015 cross-sectional parasite surveys.
2. *P. falciparum* infection incidence rate among individuals ≥ three-months-old*:* defined as the number of individuals ≥ three-months-old with a new *P. falciparum* infection (based on PCR) divided by the total person-time observed among a cohort of individuals ≥ three-months-old followed up on for a 12-month-period.
3. Total and confirmed outpatient (OPD) malaria case incidence and inpatient (IPD) malaria case incidence among all ages: defined as the number of OPD and IPD malaria confirmed and suspected cases per person per year, as ascertained from the routine rapid reporting system; facility catchment population size estimates will be used for the exposure denominator.
4. RDT positivity: defined as the proportion of all individuals tested by RDT at each round of the fMDA and MDA interventions (and among a contemporaneous sample in the control group), with a positive RDT, among the population of individuals ≥ three-months-old.

### Data collection procedures for measurement of outcomes

For all data collection procedures, written informed consent will be obtained from each participant prior to study enrollment in the trial.

#### Diagnosis of Plasmodium falciparum infections

*P. falciparum* parasite infections will be measured using RDTs, microscopy, and PCR, depending on the study outcome, as described below. The RDTs used in this trial are manufactured by (Standard Diagnostics Inc., Gyeonggi-do, Republic of Korea) under the brand name SD Bioline Malaria Antigen *P.f*, which detects the Histidine-rich protein 2 (HRP-2) antigen of *P. falciparum*. This RDT is approved by the WHO, Global Fund, and the Zambian Ministry of Health. RDTs are used across all data collection methods in this trial, including the parasite surveys, longitudinal cohort, fMDA and MDA rounds, and at health facilities in the study area. Detection of malaria parasite infections from microscopy is based on thick smears. Thick blood smears for microscopy will be used in the parasite surveys and the longitudinal cohort. PCR will be performed from capillary blood collected on dried blood spots (DBS) on filter paper, to identify low-density asexual and sexual stage parasite infections, as part of the parasite surveys and the longitudinal cohort.

#### Baseline and follow-up household parasite surveys

A cross-sectional survey of households in the high transmission season (April to May) in 2014, and again in 2015, will be conducted to obtain an unbiased sample of children under six-years-old (excluding those under one-month-old) for measuring primary outcome one (parasite prevalence). All children under six-years-old that slept in the house the night before the survey will be included in the sample. A complete enumeration and geo-referencing of all households in the study area serves as the sampling frame. To mitigate contamination between exposure groups (MDA, fMDA, and control) in the household sampling frame, sampling buffers of at least 1.5 km were derived for all HFCAs using GIS, in order to maintain an adequate distance between households in HFCAs with different exposure assignments (resulting in a gap of at least 3 km between sampled households in different HFCAs). A simple random sample of 47 households was then drawn within each of the 60 HFCAs (equal allocation sampling), to obtain the total sample size of 2,820 households in the 2014 baseline survey, after restricting the sampling frame for sampling buffers in GIS. The same sampling procedure will be used for the follow-up parasite survey in 2015.

Selected households during each survey round will be visited by trained survey data collectors that will administer a standardized survey questionnaire using mobile phones, based on the standard Malaria Indicator Survey (MIS) questionnaire [15]. Capillary blood from a finger prick from all children in selected households will be collected for detection of a malaria parasite infection by RDT, microscopy, and PCR. Children found to have a positive RDT will be treated for their infections with AL as per the Zambia National Malaria Treatment Guidelines [14].

#### Longitudinal cohort

**Fig. 4:**
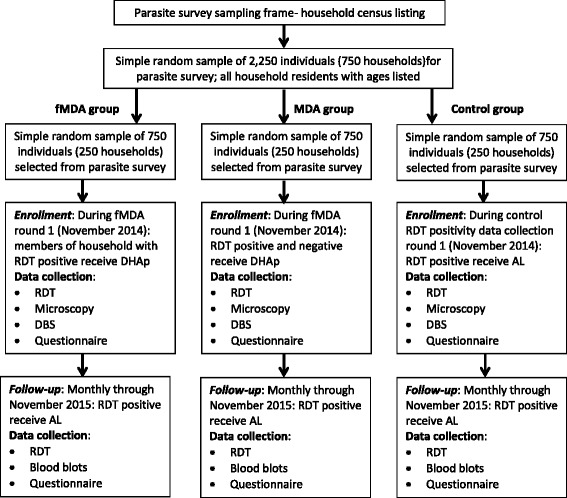
Flow chart for cohort enrollment and follow-up visit for both high and low transmission strata. *AL*: Artemether-lumefantrine; *DHAp*: Dihydroartemisinin plus piperaquine; *MDA*: Mass drug administration; *fMDA*: focal mass drug administration; *RDT*: Rapid diagnostic test; *DBL*: Dried blood spots

*P. falciparum* infection incidence will be measured by following a cohort of individuals aged three-months-old and older (and not pregnant) for measuring primary outcome indicator two, the *P. falciparum* infection incidence rate (Fig. 4). Individuals within each HFCA and treatment group, within the high and low transmission strata, will be preselected from those registered in the household roster in selected households during the baseline household parasite survey between April and May 2014. Enrollment of individuals will start during round one (November 2014) of the fMDA and MDA interventions, and concurrently among individuals during round one in control areas visited for assessing RDT positivity.

Capillary blood from a finger prick from all enrolled participants will be collected at the time of enrollment for detection of a malaria parasite infection by RDT, microscopy, and PCR. For individuals enrolled into the cohort from the round one of MDA HFCAs, all will be offered an appropriate dose of DHAp the same as those in the MDA study, irrespective of RDT result. For individuals enrolled into the cohort from the round one fMDA HFCAs, those testing positive by RDT or in households with any member testing positive by RDT will be offered an appropriate dose of DHAp the same as those in the fMDA study. Individuals enrolled into the cohort in control HFCAs testing positive by RDT will receive age and weight appropriate formulations of AL, as per standard of care guidelines [14].

Enrolled individuals within the MDA, fMDA, and control HFCAs will then be followed up on every four weeks from enrollment date for collection of blood for an RDT, microscopy, and DBS for PCR, until the study’s conclusion in November 2015. Any individual testing positive for circulating malaria antigen by RDT during any visit after enrollment will receive treatment of age- and weight-appropriate doses of AL, as per standard of care guidelines [14].

### Routine health information on confirmed and suspected malaria cases

Data on aggregate clinical and laboratory confirmed malaria cases and total outpatients seen are submitted on a weekly basis, through a standardized forma on data-enabled cell phones, using the District Health Information System 2 (DHIS2) rapid reporting system, from all 60 health facilities in the study area. Data from this source are accessible via a web server with password protection. This system was established in 2011 and 2012 and has been running in the catchment areas ever since. Time series data by facility week will be collated from this system to provide a passive incidence-based data set for evaluation of the impact of the MDA and fMDA interventions, as compared to a control of no mass treatment, on primary outcome three. Confirmed and total case counts will be standardized by the estimated mid-year populations of each HFCA.

### Rapid diagnostic test positivity during campaign rounds

As described above under the MDA and fMDA intervention descriptions, all individuals aged three-months-old and older in these HFCAs will be tested by RDT for detecting malaria parasite infections; this will then be used for ascertaining primary outcome four (RDT positivity) in each HFCA. RDT positivity in control areas during each of the two rounds of the MDA and fMDA interventions in 2014, and again in 2015, will be obtained through comparing RDT positivity among individuals tested as part of the monthly control cohort during the months when the MDA and fMDA rounds occur.

#### Cost data

Cost data collection will be coupled with the study in order to provide numerator estimates for cost-effectiveness calculations. Cost data collection will be provider focused and will not require collection of data from intervention participants or research subjects. Data on the cost of inputs to the intervention will be collected using a review of program records and reports, invoices, budgets, expenditure reports, and interviews with intervention implementers. Interviews will be focused on resource use during the implementation of the study interventions. Where direct estimates of unit costs for inputs are not available through the above methods, data on costs will be supplemented with secondary source data, such as is available from the WHO-CHOICE database, the National Bank of Zambia, the World Bank, the International Monetary Fund, or other published literature. Economic costs will be estimated, meaning that costs from donated inputs will also be valued.

#### Sample size and statistical power

The maximum number of cluster units available was 60, split evenly with 10 clusters per MDA, fMDA, and control groups in the high and low transmission strata. Sample size was derived using a formula for CRCTs developed by Hayes and Moulton [16]. Sample sizes are estimated to assess statistical differences comparing the four primary outcomes for the MDA group against no intervention (control), and for the fMDA group against no intervention (control).

#### Baseline and follow-up household parasite surveys

##### High transmission strata

The coefficient of variation (CV) was estimated using data from a previous study of the 2012 MTAT-AL in the study area (CV = 0.31). Based on the number of clusters available in the high transmission strata (n = 10 in each of the three treatment groups, total of 30 clusters), a statistical power of 80 %, a probability of committing a type two error of 5 % (one-tailed test), and simple random sampling used to obtain the sample of children in each household survey, a sample size of 39 children under-years-old per HFCA per treatment group will be needed to detect 12 % absolute detectable difference in parasite prevalence between any two groups, from a baseline of 30 % (a 40 % relative difference, going from 30 to 18 % parasite prevalence). This detectable difference equates to a crude odds ratio of 0.51. With survey non-response assumed to be 20 %, a sample size of 470 children per treatment group (47 children per cluster), totaling 1,410 children under six-years-old per survey round will be sought (Additional file 1: Table S2). This sample size equates to a sample size of 1,410 households sampled per survey round.

##### Low transmission strata

Based on the number of clusters available in the low transmission strata (n = 10 in each of the three treatment groups, total of 30 clusters), the observed CV (CV = 0.41), a statistical power of 80 %, a probability of committing a type two error of 5 % (one-tailed test), and simple random sampling used to obtain the sample of children in each household survey, a sample size of 39 children under six-years-old per HFCA per treatment group will be needed to detect 6 % absolute detectable difference in parasite prevalence between any two groups from a baseline of 10 % (a 60 % relative difference going from 10 to 4 % parasite prevalence). This detectable difference equates to a crude odds ratio of 0.38. With survey non-response assumed to 20 %, a sample size of 470 children per treatment group (47 children per cluster), totaling 1,410 children under six-years-old per survey round will be sought (Additional file 1: Table S2). This sample size equates to a sample size of 1,410 households sampled per survey round.

#### Longitudinal cohort

##### High transmission strata

The CV for infection incidence was based on estimates of OPD confirmed case incidence for HFCAs for 2012 (CV = 0.50). Based on the 30 HFCAs available in the high transmission strata, a statistical power of 80 %, a probability of committing a type two error of 5 % (one-tailed test), a sample size of 25 individuals aged three-months-old and older per HFCA (cluster) per treatment group will be needed to detect an absolute detectable difference in 12-month cumulative parasite infection incidence between any two groups from an assumed baseline of 1.8 infections per person per year to 0.91 infections per person per year (a 49 % relative reduction). With a loss to follow-up rate assumed to be 33 %, a sample size of 375 individuals per treatment group (38 per HFCA) will be enrolled and followed up on monthly, totaling 1,125 individuals aged three-months-old and older enrolled in the cohort study in the high transmission strata (Additional file 1: Table S3).

##### Low transmission strata

The CV for infection incidence was based on estimates of OPD confirmed case incidence for HFCAs in 2012 (CV = 0.50). Based on the 30 HFCAs available in the low transmission strata, a statistical power of 80 %, a probability of committing a type two error of 5 % (one-tailed test), a sample size of 27 individuals aged three-months-old and older per HFCA (cluster) per treatment group will be needed to detect absolute detectable difference in parasite infection incidence between any two groups from an assumed baseline of 0.6 infections per person per year to 0.28 infections per person per year (a 53 % relative reduction). With a loss to follow-up rate assumed to be approximately 33 %, a sample size of 375 individuals per treatment group (38 per cluster) will be enrolled and followed up on monthly, totaling 1,125 individuals enrolled in the cohort study in the low transmission strata (Additional file 1: Table S3).

#### Rapid diagnostic test positivity during campaign rounds

It is estimated based on the population sizes of the MDA and fMDA HFCAs, a sample size exceeding 50,000 individuals aged three-months-old and older in each group (approximately 5,000 per HFCA) will be achieved. As described above, a sample size of 750 individuals aged three-months-old and older in the cohort in the control HFCAs will provide contemporaneous measure of RDT positivity in areas without intervention rounds (n = 375 individuals in the high transmission strata and 375 individuals in the low transmission strata). This sample size in areas of high transmission will provide 80 % statistical power to detect a 33 % difference (an absolute decrease from 30 to 20 %; odds ratio of 0.58) in RDT positivity between the control group and either of the MDA groups, assuming a baseline prevalence of 30 %, a CV = 0.31, 10 HFCAs in each treatment group, and a probability of committing a type two error of 5 % (one-tailed test). This sample size in areas of low transmission will provide 80 % statistical power to detect a 50 % difference (an absolute decrease from 10 to 5 %; odds ratio of 0.47) in RDT positivity between the control group and either of the MDA groups, assuming a baseline prevalence of 10 %, a CV = 0.41, 10 HFCAs in each treatment group, and a probability of committing a type two error of 5 % (one-tailed test).

### Statistical analysis plan

The primary analysis for estimating the treatment effect between MDA, fMDA, and control HFCAs for primary outcome one (parasite prevalence) and two (parasite infection incidence rates) will be based on intention to treat at the individual level for infections measured by RDT and PCR. The analysis of parasite prevalence will use a logistic or log binomial model, while the analysis for infection incidence will use a Poisson or negative binomial model. Models will include a random intercept at the HFCA level (randomization unit), fixed effect for intervention exposure, time period (pre- and post-intervention), plus other potential confounding factors. Standard errors will be estimated, with the addition of repeated measures among individuals.

Data for total and confirmed OPD and IPD malaria case incidence (outcome three) among all ages will be analyzed on an intention-to-treat basis. Routine data on case counts will be analyzed using a Poisson or negative binomial model, with random intercepts at the HFCA level. The models will include a fixed effect for study arm and time period (pre- and post-intervention), plus other potential confounding factors.

Differences in prevalence measures (RDT positivity) at each round will be assessed using an *X*^2^ test, as well as logistic regression models to account for potential confounding factors, on an intention-to-treat basis. Logistic models will include a random intercept for HFCA, as well as fixed effects for other potential confounding factors. Trends in parasite prevalence across rounds, with the inclusion of the control group, will then allow rapid assessment of the effect of the fMDA and MDA interventions over time, after removing the seasonality effect.

Standard methods for collecting cost data for the specific components of the work will be used to then summarize intervention costs. Cost-effectiveness calculations will be undertaken utilizing cost data collected as above, as well as the findings of the study evaluation on the effectiveness of the intervention. The primary cost-effectiveness outcome will be the calculation of the incremental cost effectiveness ratios (ICERs) comparing the cost-effectiveness of MDA to the standard of care, and the ICER of fMDA alone versus standard of care. Secondary outcomes will include the ICER of MDA versus fMDA alone, and the stratified analysis of all three previously listed outcomes by high and low prevalence settings. Further details of the cost and cost-effectiveness analysis are presented in the Additional file 1.

### Quality control and secondary studies

#### Mass drug administration and focal mass drug administration intervention coverage

The population coverage of the MDA and fMDA interventions will be measured in two ways at each intervention round. Operational program coverage is defined as the proportion of individuals aged three-months-old and older, and households visited, offered the MDA and fMDA interventions within the target areas. Effective program coverage is defined as the proportion of individuals (aged three-months and older) that agreed to participate in the MDA and fMDA interventions among all individuals eligible to participate in the intervention in the target population.

The operational coverage will be estimated as the percent of the population that received a visit from the mass treatment teams to offer the MDA or fMDA interventions, among those eligible for inclusion. This will be obtained from a combination of MDA and fMDA program data and census enumeration data. Additionally, the proportion of individuals accepting the MDA and fMDA interventions aged three-months-old and older, among those eligible for inclusion in the intervention, will provide an estimate of the effective coverage of each program, as outlined below. Data for the denominator of individuals and households targeted for the intervention will be ascertained from the household enumeration for the sampling frame. Individual, household, and community level factors associated with coverage will be assessed using mixed effects logistic regression.

#### Participant adherence to dihydroartemisinin and piperaquine

A total of 150 individuals enrolled in the cohort study at round one, with a positive RDT in the MDA (n = 75) and fMDA (n = 75) rounds, plus another 150 individuals enrolled in the cohort study at round one, with a negative RDT in the MDA round, will be used to assess the level of adherence with the prescribed DHAp antimalarial regimen. Further details of the primary outcomes, sample size calculations, data collection methods, and the statistical analysis plan for this sub-study are provided in the Additional file 1.

#### Plasmodium falciparum parasite clearance following dihydroartemisinin and piperaquine

A total of 150 individuals enrolled in the cohort study at round one, with a positive RDT in the MDA (n = 75) and fMDA (n = 75) rounds, will be used to assess parasite clearance following DHAp. Further details of the primary outcomes, sample size calculations, data collection methods, and the statistical analysis plan for this sub-study are provided in the Additional file 1.

#### Acceptability of dihydroartemisinin and piperaquine in the community

The assessment of community acceptability of the MDA and fMDA interventions will be ascertained using a mixed methods approach. Acceptability of the interventions will be assessed quantitatively during the baseline and follow-up parasite surveys among WRA. Focus groups will also be used to qualitatively assess community perceptions of the acceptability of the MDA and fMDA interventions, including community members who consented and participated, as well as those who refused. Further details of the acceptability study are provided in the Additional file 1.

### Study timeline

**Fig. 5:**
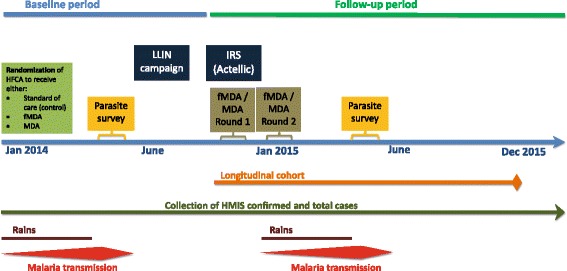
Study timeline for major activities. *MDA*: mass drug administration; *fMDA*: Focal mass drug administration; *LLIN*: Long-lasting insecticide-treated nets; *IRS*: Indoor residual spraying; *HFCA*: Health facility catchment area; *HMIS*: Health management information system

Figure 5 shows the timeline for the major activities for the primary study of evaluating the impact of the MDA and fMDA interventions on reducing malaria morbidity. The study started with the randomization of HFCA in early 2014. The baseline parasite survey was conducted between April and May of 2014 during the peak malaria transmission season. The first MDA and fMDA rounds were conducted between November and December 2014, with round two scheduled for February 2015. The longitudinal cohort started at the same time as the first MDA and fMDA round in November 2014; the cohort will continue monthly follow-up through November 2015. Routine data on confirmed OPD malaria cases started in January 2014 and will continue through 2015. The follow-up parasite survey is scheduled to take place between April and May 2015, during the peak malaria transmission season.

### Ethical approval

Ethical approval in Zambia for the primary and secondary studies has been obtained from the Research Ethics Committee (REC) at the University of Zambia (study reference 007-03-14) and the Zambian Medicines Regulatory Authority (trial number CT 033/12). Ethical approval was also obtained from the Tulane Institutional Review Board (IRB; study number 59208573) and Western IRB (covering PATH-MACEPA; study number 1146551).

## Discussion

In line with the goals of the Zambia National Malaria Strategic Plan, robust comparisons of the effectiveness and cost-effectiveness of available mass treatment approaches, including MDA and fMDA interventions using DHAp, are needed. Toward that end, this study will assess the relative effectiveness of two mass treatment approaches at reducing the malaria burden beyond already implemented interventions of LLINs and IRS. The relative effectiveness of MDA and fMDA with DHAp will be established against a comparison of no mass treatment (standard of care) in areas with higher and lower malaria parasite prevalence using a community randomized control trial. The findings of this study will provide the Zambia NMCC and their partners with guidance on the most effective and cost-effective strategies to further reduce the size of the malaria parasite reservoir, malaria transmission, and the incidence of clinical disease in southern Zambia, and will hopefully show evidence of local transmission interruption.

The strengths of this trial include the fact that a rigorous study design (a CRCT) will be used to assess the impact of the MDA and fMDA interventions, as compared to a control of no mass treatment (standard of care). The study is also powered to assess differences between each treatment group (MDA and fMDA) and the control group, within areas of high and low transmission, with 80 % statistical power. The study uses robust measures of malaria parasite infection at the point of contact using RDTs, as well as PCR, for detecting low-density parasite infections, as part of the parasite surveys and the longitudinal cohort. The study also uses multiple methods for measuring malaria-related outcomes, including malaria parasite infection prevalence ascertained from a pre- and post-parasite survey, malaria parasite infection incidence using a longitudinal cohort of individuals selected randomly from within each HFCA, and confirmed malaria case incidence throughout the study using routine health management information system (HMIS) data in the study area. Buffer areas between contiguous HFCAs with different interventions are being used to limit contamination and misclassification bias. Measures of the quality of the implementation of the MDA and fMDA interventions will be obtained to help with the interpretation of results; these include MDA and fMDA population coverage, adherence to DHAp, parasite clearance after DHAp, community perceptions and acceptability of the MDA and fMDA interventions, and multiple measures of operational coverage. Lastly, a rigorous analysis plan will be followed, for assessing the impact of each intervention compared to a control group, while accounting for potential contextual and confounding factors within random-effect regression models.

Noted weaknesses of this trial include a less than ideal number of community randomization units (N = 60), especially when stratified by high (n = 30) and low (n = 30) transmission strata. The sample size within the high and low transmission strata will only allow the detection of approximately a 50 % reduction in parasite prevalence and infection incidence between intervention groups, with 80 % statistical power; the threat of a type two error is therefore higher than ideal. The primary analysis will be based on the intention-to-treat principle; there is potential for misclassification of exposure using this approach. Secondary analysis using a per-protocol approach will be used mitigate any misclassification of exposure. And lastly, there is always the potential for error in the measurement of outcomes, exposure to the interventions, and potential confounding factors in such a large and complex trial. We have gone to considerable length to try and minimize such error through the use of electronic data capture systems in the field and robust diagnostic methods.

## Trial status

The study is ongoing. The baseline parasite survey was completed in April to May 2014. The first round of the MDA and fMDA intervention was completed in November to December 2014; the longitudinal cohort started at the same time. The second round of the MDA and fMDA intervention is scheduled for February 2015 (concluding in mid-March 2015). The follow-up parasite survey is scheduled for April to May 2015, while monthly longitudinal follow-up is scheduled to continue through November 2015.

## Additional file

Additional file 1:
**Web appendix containing supporting information.** (DOCX 60 kb)

## Abbreviations

AL: Artemether lumefantrine
CRCT: Cluster randomized control trial
DHA: Dihydroartemisinin
DHAp: Dihydroartemisinin plus piperaquine
DOT: Directly observed therapy
fMDA: Focal mass drug administration
HFCA: Health facility catchment area
ICER: Incremental cost effectiveness ratio
IPD: Inpatient
IRB: Institutional review board
IRS: Indoor-residual spraying
LLIN: Long-lasting insecticide-treated mosquito net
MDA: Mass drug administration
NMCC: National Malaria Control Center
OPD: Outpatient
RDT: Rapid diagnostic test
REC: Research ethics committee
PCR: Polymerase chain reaction
WHO: World Health Organization
WRA: Women of reproductive age
