# Supplementary material for: Assessing the effectiveness of household-level focal mass drug administration and community-wide mass drug administration for reducing malaria parasite infection prevalence and incidence in Southern Province, Zambia: study protocol for a community randomized controlled trial
Source: Trials. 2015 Aug 13;16:347. doi: 10.1186/s13063-015-0862-3 (PMC4535296; doi:10.1186/s13063-015-0862-3)
Supplement: Additional file 1: — Web appendix containing supporting information. (DOCX 60 kb) [file 13063_2015_862_MOESM1_ESM.docx]

**Additional file 1**

**Web appendix containing supporting information**

**Details of cost and cost-effectiveness analysis**

Standard methods for collecting cost data for the specific components of the work will be used to then summarize intervention costs. Cost-effectiveness calculations will be undertaken utilizing cost data collected as above as well as the findings of the study evaluation on the effectiveness of the intervention. The primary cost-effectiveness outcome will be the calculation of the Incremental Cost‑Effectiveness Ratios (ICER) comparing the cost-effectiveness of mass drug administration (MDA) to the standard of care (SoC) and the ICER of focal mass drug administration (fMDA) alone vs. SoC. Secondary outcomes will include the ICER of MDA vs. fMDA alone, and the stratified analysis of all three previously listed outcomes by high and low prevalence settings. Further details of the cost and cost-effectiveness analysis are presented in this appendix. The effect estimates will be derived in primary analysis using the effectiveness estimates on incidence available from the cohort study. Secondary effect estimates will be derived from passive HF incidence data. The general form of the equation for the calculation of ICERs is as follows:

$ICER=\frac{C_{MDA+CP}-C_{SoC}}{E_{MDA+CP}-E_{SoC}}$.

Where CMDA is the total cost per person of providing MDA to the study population, CSoC is the total cost per person of providing the SoC intervention to the study population, EMDA is the effect in terms of infections averted per person year in the MDA arm and ESoC is the effect in terms of infections averted per person year in the SoC arm. In the SoC comparisons both CSoC and ESoC are generally expected to be zero, and thus in these comparisons the above equation will reduce to the simple formulation:

$ICER=\frac{C_{MDA+CP}}{E_{MDA+CP}}$.

**Assessment of adherence and parasite clearance with the dihydroartemisinin – piperaquine (DHAp) regimen given during the fMDA and MDA interventions**

Directly observed treatment (DOT) will be used to maximize adherence with the prescribed DHAp among all individuals in households with a member having a positive rapid diagnostic test (RDT) participating in the fMDA and MDA interventions. Obtaining high adherence with taking a full course of DHAp is critical to maximizing the effectiveness of the fMDA and MDA interventions for several important reasons. First, if a full course of DHAp is not taken, the malaria parasites may not be cleared and the individual will remain infected and will also remain infective for onward transmission. And second, the chemoprevention properties of the fMDA and MDA intervention rely on having as much DHAp in the population as possible to prevent individuals from being infected from an infectious mosquito bite.

***Implementation design***

Individuals ≥three months old enrolled in the cohort study at round 1 in the fMDA and MDA areas will be used to: 1) assess the level of adherence with the prescribed DHAp antimalarial regimen; and 2) assess the parasite clearance following DHAp. The study is nested within the cohort study enrollment at round 1 of the fMDA and MDA interventions.

***Primary outcome measures***

1. *Proportion of patients fully adherent with prescribed drug regimen*: defined as the proportion of individuals ≥three months old enrolled in the cohort study that self-report taking their prescribed antimalarial tablet on the first, second and third day of treatment (DHAp is given one tablet per day for three days), among individuals enrolled in the cohort study who were assigned a treatment regimen at round 1 in the fMDA and MDA interventions (2014).
2. *Proportion of patients with physical evidence of full adherence with the prescribed drug regimen*: defined as the proportion of individuals ≥three months old enrolled in the cohort study that can show an interviewer the used blister pack of the prescribed antimalarial regimen according to the appropriate treatment schedule, among individuals enrolled in the cohort study who were assigned a treatment regimen at round 1 in the fMDA and MDA interventions (2014).
3. *Proportion of patients who refused treatment*: defined as the proportion of individuals ≥three months old living in households visited by the fMDA and MDA study teams that refused treatment, among those eligible for treatment regimen (RDT positive in fMDA areas and RDT positive and negative in MDA areas).
4. *Proportion of individuals who cleared parasite infection*: defined as the proportion of individuals ≥three months old with a negative microscopy result on day 7 (seven days after initial dose of DHAp), among individuals ≥three months old enrolled in the cohort study at round 1 in the fMDA and MDA interventions (2014) that had a positive microscopy result and given DHAp on day 0.

***Sample size***

The sample sizes for the adherence sub-study among those individuals enrolled in the cohort study positive for a parasite infection at round 1 of the fMDA and MDA interventions are outlined in SI Table 4. Assuming parasite prevalences of 25% and 5% in the high and low transmission strata, respectively, there should be a total of 150 individuals ≥three months old with a parasite infection (positive RDT) given DHAp for inclusion in this sub-study to assess adherence and parasite clearance. An additional 150 individuals from the MDA intervention areas across the high and low transmission strata with negative RDTs given DHAp for chemopreventive purposes will also be included in this sub-study to assess the adherence to the DHAp regimen. For adherence outcomes 1 and 2 among those RDT positive (fMDA and MDA intervention) and RDT negative (MDA intervention), a sample size of 150 RDT positive individuals and 150 RDT negative will allow the estimation of adherence with a 95% confidence interval ±12%, assuming adherence of 80%, a probability of committing a type-1 error of 5% (two sided test), 10% refusal, and substantial interclass correlation at the household and health facility catchment area (HFCA) level, represented by a design effect of 3.

For the parasite clearance outcome (outcome 4) among those RDT positive (and subsequently confirmed by microscopy), a sample size of 150 individuals will allow the estimation of parasite clearance with a 95% confidence interval ±8.5%, assuming parasite clearance of 90%, a probability of committing a type-1 error of 5% (two sided test), 10% refusal, and substantial interclass correlation at the household and HFCA level, represented by a design effect of 3.

***Data Collection***

DHAp adherence

All individuals testing positive by RDT of the 1,500 individuals (500 households) enrolled into the cohort study during round 1 of the fMDA and MDA interventions will be eligible for inclusion in the adherence assessment, which based on parasite prevalence from past MTAT rounds should yield at least 150 individuals ≥three months old (within approximately 75-150 households depending on the number positive per household) that test positive and receive DHAp (Figure 6). Of the 750 individuals enrolled into the cohort in the MDA intervention group, approximately 425 individuals ≥three months old with a negative RDT who receive DHAp, from which a sample of 150 (75-100 households) will be selected for assessing adherence among those in the chemoprevention group (i.e. given DHAp although RDT negative).

Following informed consent during the cohort enrollment during round 1 of the fMDA and MDA interventions (June–July 2015), the 75 households selected for inclusion will be visited on day 3 (first dose of DHAp on day 0; day 3 is on day following last dose of DHAp) by trained data collection teams. All individuals will have a standardized questionnaire administered to assess whether they took the assigned doses of DHAp by number of doses and by day scheduled, in full and according to the dosing schedule and timing prescribed (Appendix 14). This data will be self-reported by the participant if aged ten years or older, or by the caregiver for those nine years and younger. In addition, participants will be asked to produce the remaining blister pack for the medication so that study teams can assess the fraction of pills from the DHAp regimens which have actually been removed from the blister packaging. Study teams will enter data on a standardized form to capture data about blister pack retention and removal of medicine from the blister packs. Additionally, hypothesized risk factors for non‑adherence, including study transmission strata, RDT test result, intervention round, age, sex, education level, household residence, household socioeconomic status, recent diagnosis of malaria, and presence of other illnesses will be collected using standardized questionnaires.

Parasite clearance following DHAp

The same consenting individuals outlined above for the adherence study testing positive by RDT among the 1,500 individuals (500 households) enrolled into the cohort study during round 1 of the fMDA and MDA interventions will be included in the parasite clearance assessment, which, based on parasite prevalence from past MTAT rounds, should yield at least 150 individuals ≥three months old within about 75 households that test positive and receive DHAp (SI Figure 1). The same trained data collection teams used for the adherence data collection will also be used for the parasite clearance data collection. On day 3 at the same visit for adherence, blood slides and two drops of blood on filter paper will be collected. A follow-up visit on day 7 (seven days after first treatment on day 0) will be conducted with blood slides and two drops of blood on filter paper collected for each consenting individual. Slides will be stored in slide boxes and transported to a central location for reading. Participants identifying information will be replaced with a unique identifier on each slide. All appropriate universal and site-specific safety precautions will be used in handling blood slides and RDTs while in laboratories. Survey workers will be trained in the proper storage and handling of slides prior to initiation of field work. Individuals with a positive day 7 blood slide will be followed up and treated with an age and weight appropriate dose of artemether-lumefantrine (AL), as is standard of care in Zambia. Blood blots on filter paper will be analyzed by PCR for adjusting parasite clearance measures for excluding new infections.

***Statistical analysis plan***

The proportion of patients fully compliant with the prescribed drug regimen will be analyzed by comparing the prevalence of full adherence with study drug regimen among participants by fMDA and MDA groups. Statistical testing will utilize the χ2-test for prevalence differences between groups. Logistic regression will be used to compare various individual and household factors including transmission strata, malaria RDT result, age, fever, presence of any adverse events in the individual or the household, and other potential determinants of adherence. HFCA and household will be included as random effects.

Proportion of patients with physical evidence of full adherence with the prescribed drug regimen will be analyzed by comparing the prevalence of fully completed blister packs consistent with full adherence to the DHAp regimen among participants by fMDA and MDA intervention group. Statistical testing will utilize the χ2-test for prevalence differences between arms. Logistic regression will be used to compare various individual and household factors including transmission strata, malaria RDT result, age, fever, presence of any adverse events in the individual or the household, and other potential determinants of adherence. HFCA and household will be included as random effects.

The proportion of patients who refused treatment will be analyzed by comparing the number of individuals who reported refusing treatment to the number of individuals that were eligible for treatment among participants in fMDA and MDA intervention groups. Statistical testing will utilize the χ2-test for prevalence differences between groups. Logistic regression will be used to compare various individual and household factors including transmission strata, malaria RDT result, age, fever, presence of any adverse events in the individual or the household, and other potential determinants of treatment refusal. HFCA and household will be included as random effects.

The proportion of individuals who cleared parasite infection will be analyzed by comparing the proportion of individuals without parasites detected from microscopy on day 7, among all those receiving DHAp for a parasite infection on day 0 established by microscopy. Logistic regression will be used to compare various individual and household factors including transmission strata, age, fever, presence of any adverse events in the individual or the household, and other potential determinants of treatment refusal. HFCA and household will be included as random effects.

**Acceptability of DHAp in the community**

***Implementation procedures***

The assessment of community acceptability of the fMDA and MDA interventions will be ascertained using a mixed methods approach. Acceptability of the interventions will be assessed quantitatively during the baseline and follow-up parasite surveys among women of reproductive age (WRA). Focus groups will also be used to qualitatively assess community perceptions of the acceptability of the fMDA and MDA interventions, including community members who consented and participated as well as those who refused. Within both groups, perceived barriers to participating in the interventions will also be assessed. In particular, the acceptability of taking medicines for malaria to clear parasites and provide prophylactic protection against malaria among those in the MDA intervention will be assessed quantitatively and qualitatively. Focus group and semi-structured interviews of community health workers (CHWs) will be used to assess barriers to adherence, experience in implementing the interventions, and the acceptability of the interventions among community members.

***Primary outcomes***

The primary qualitative outcome for this study will be the common perceived beliefs on why community members choose to participate and choose not to participate in the fMDA and MDA interventions. The primary quantitative outcome will be the proportion of survey respondents who strongly disagree, disagree, are ambivalent, agree, and strongly agree on the importance of community mass treatment and the provision of prophylactic protection.

***Sample size***

For the primary quantitative indicator above, the sample of 2,700 households across the fMDA and MDA interventions at the baseline (2014) and again at follow-up (2015) survey rounds will be used. This is expected to provide a sample size of at least 4,000 individuals at each round, which is sufficient to estimate the proportion of participants who agree that the MDA and fMDA intervention are important (agree and strongly agree) with ±5% intervals with the probability of committing a type-1 error of 5% (1-tailed test).

For the qualitative indicator above, convenience sampling will be used to 1) identify community members in the fMDA and MDA intervention areas who agreed to participate and who refused to participate in the 2014 and 2015 fMDA and MDA interventions (e.g. to take the prescribed antimalarial regimens) and 2) community health workers and other health program staff implementing the interventions.

Approximately 100 individuals in the fMDA and MDA interventions (50 in the fMDA and 50 in the MDA during the 2014 and 2015 rounds) will be targeted for qualitative data collection using focus group interviews. Approximately 30 individuals identified as CHWs or health facility staff will additionally be targeted for qualitative data collection during focus group interviews. A total sample size of 130 men and women ≥18 years old will be targeted in 2014 and again in 2015 (total n = 260).

***Data collection***

Informed consent will be obtained to participate specifically in the semi-structured and focus group interviews. It is expected that five focus groups of ten community members will be conducted in the fMDA (n = 5 groups and 50 community members) and MDA (n = 5 groups and 50 community members) groups after the third fMDA/MDA round in each year, totaling 200 community members. Standardized interview and discussion guides will be established using pre-testing and best practices in qualitative research. Trained interviewers will be used to conduct the interviews in the local languages. An additional two focus groups of ten community health workers will be conducted after the third survey round in each year (n = four groups and 40 health workers).

Semi-structured interviews will be conducted with community health workers and other health professionals who implemented the interventions. These interviews will explore constraints and problems related to all the topics identified from the quantitative survey, field experience implementing the fMDA and MDA interventions, and barriers to implementation of malaria control efforts. They are also important in order to involve providers in the study.

A specific acceptability module with Likert scale questions will be added to the Parasite Survey questionnaire and collected as part of the Parasite Surveys in 2014 and 2015, as described in detail above.

***Analytic plan***

The interviews will be conducted in local languages and field notes will be written during the interview. Each afternoon, fieldworkers will return to a central location and work with the local investigator to systematically record field notes in English (using the ‘Fair Notes’ data capture method). These notes will be transcribed or entered directly into MS Word. dtSearch 6.0 © (dtSearch Corp.) will be used to index and search these notes as data collection proceeds. Analysis will identify thesauri of common themes and interpretations and will summarize each section of the assessment guide. These summaries will be compared to summaries maintained by the fieldworkers. Although supervision will take place each day, a special meeting with the fieldworkers will be held to review findings from data and to follow up with special issues.

Pile sorting of responses to questions surrounding the testing and treatment of adults and children will be performed for group interviews, with most common and important barriers to fully participating in the fMDA and MDA interventions identified.

For the quantitative analysis, Likert scale data will be grouped as needed using Cronbach’s coefficient alpha. Data will then be categorized and analyzed using descriptive statistics, χ2s and logistic regression.

**SI Figure 1** Flow chart for the adherence and parasite clearance sub-study for both high and low transmission strata.

**MDA group**

**MDA group**

**fMDA group**

All RDT positive individuals ≥3m enrolled in the cohort study included (n=75)

All RDT positive individuals ≥3m enrolled in the cohort study included (n=75)

Sample of RDT negative individuals ≥3m enrolled in the cohort study included (n=150)

from parasite survey

***Data collection***

Day 3:

- DHAp adherence questionnaire

***Data collection***

Day 3:

- DHAp adherence questionnaire
- Microscopy
- Blood blots

Day 7

- Microscopy
- Blood blots

***Data collection***

Day 3:

- DHAp adherence questionnaire
- Microscopy
- Blood blots

Day 7

- Microscopy
- Blood blots

***Outcomes***

- DHAp adherence among RDT− (outcomes 1-2)

***Outcomes***

- DHAp adherence among RDT+ (outcomes 1-2)
- Parasite clearance day 7 (outcome 4)

***Outcomes***

- DHAp adherence among RDT+ (outcomes 1-2)
- Parasite clearance day 7 (outcome 4)

**SI Table 1** Age-based dosing guidelines used for administering DHAp

| **Age** | **Weight (kg)** | **DHAP (40/320mg)** | | |
| --- | --- | --- | --- | --- |
|  | **(kg)** | **pill count per day** | **mg** | **mg** |
|  |  |  | **40** | **320** |
| 0–3 months | <5 |  |  |  |
| 3–12 months | 8 | 0.5 | 2.50 | 20.00 |
| 1 year | 10 | 1 | 4.00 | 32.00 |
| 2 | 11 | 1 | 3.64 | 29.09 |
| 3 | 15 | 1 | 2.67 | 21.33 |
| 4 | 15 | 1 | 2.67 | 21.33 |
| 5 | 18 | 1 | 2.22 | 17.78 |
| 6 | 21 | 1 | 1.90 | 15.24 |
| 7 | 23 | 1 | 1.74 | 13.91 |
| 8 | 25 | 2 | 3.20 | 25.60 |
| 9 | 28 | 2 | 2.86 | 22.86 |
| 10 | 30 | 2 | 2.67 | 21.33 |
| 11 | 35 | 2 | 2.29 | 18.29 |
| 12 | 39 | 2 | 2.05 | 16.41 |
| 13 | 45 | 2 | 1.78 | 14.22 |
| 14 | 50 | 3 | 2.40 | 19.20 |
| ≥15 years | >50 | 3 | <2.4 | <19.2 |
|  |  | **AVG mg/kg/day** | **2.57** | **20.57** |

**SI Table 2** Sample size for children <6 years old and households, for detecting differences in parasite prevalence between treatment groups at each survey round

| **Strata** | **Treatment group** | **Number clusters / HFCAs** | **Total sample of children per cluster for detecting ∆*** | **Total n required**  **(children / households)*** |
| --- | --- | --- | --- | --- |
| **High malaria transmission (approximately >10% parasite prevalence)** | fMDA-DHAp | 10 | 47 | 470 / 470 |
|  | MDA-DHAp | 10 | 47 | 470 / 470 |
|  | Control | 10 | 47 | 470 / 470 |
| **Low malaria transmission (approximately ≤10% parasite prevalence)** | fMDA-DHAp | 10 | 47 | 470 / 470 |
|  | MDA-DHAp | 10 | 47 | 470 / 470 |
|  | Control | 10 | 47 | 470 / 470 |
| **Total** |  | **60** |  | **2,820 / 2,820** |

*Accounting for loss-to-follow-up and survey non-response

**SI Table 3** Sample size for individuals >3 months old for detecting differences in parasite infection incidence between treatment groups over a 12 month period (each individual = 1 person-year)

| **Strata** | **Treatment group** | **Number clusters / HFCAs** | **Total sample per cluster for detecting ∆ / households*** | **Total n required / households*** |
| --- | --- | --- | --- | --- |
| **High malaria transmission (approximately >10% parasite prevalence)** | fMDA-DHAp | 10 | 38 / 13 | 375 / 125 |
|  | MDA-DHAp | 10 | 38 / 13 | 375 / 125 |
|  | Control | 10 | 38 / 13 | 375 / 125 |
| **Low malaria transmission (approximately ≤10% parasite prevalence)** | fMDA-DHAp | 10 | 38 / 13 | 375 / 125 |
|  | MDA-DHAp | 10 | 38 / 13 | 375/ 125 |
|  | Control | 10 | 38 / 13 | 375/ 125 |
| **Total** |  | **60** |  | **2,250 / 750** |

*Accounting for 20% loss-to-follow-up

**SI Table 4** Sample size for individuals ≥3 months for quantifying DHAp adherence and day 7 parasite clearance, among individuals with positive RDT given DHAp enrolled in cohort study at Round 1 2014.

| **Strata** | **Treatment group** | **Number clusters / HFCAs** | **Total sample per cluster for detecting ∆*** | **Number individuals/ households enrolled in cohort*** | **Number individuals for activity 2 RDT+ and given DHAp** |
| --- | --- | --- | --- | --- | --- |
| **High malaria transmission (approximately >10% parasite prevalence)**** | fMDA-DHAp | 10 | 25 | 250 / 125 | 62 |
|  | MDA-DHAp | 10 | 25 | 250 / 125 | 62 |
| **Low malaria transmission (approximately ≤10% parasite prevalence)***** | fMDA-DHAp | 10 | 25 | 250 / 125 | 13 |
|  | MDA-DHAp | 10 | 25 | 250 / 125 | 13 |
| **Total** |  | **60** |  | **1,000 / 500** | **150** |

Another 150 individuals 3 months – 20 years old RDT negative given DHAp in the MDA intervention area will also be included to assess adherence.

*Accounting for 20% loss-to-follow-up; **Assuming 25% parasite prevalence at first fMDA/MDA round; ***Assuming 5% parasite prevalence at first fMDA/MDA round
